# Multi-disciplinary simulation training improves confidence in managing physical health emergencies on the psychiatric inpatient ward

**DOI:** 10.1192/j.eurpsy.2026.10960

**Published:** 2026-07-31

**Authors:** D. Di Francesco, A. Colombo-Sansom

**Affiliations:** 1 Sussex Partnership NHS Foundation Trust, Eastbourne; 2 Sussex Partnership NHS Foundation Trust, Brighton, United Kingdom

## Abstract

**Introduction:**

We have developed a multi-disciplinary simulation training programme to improve management of physical health emergencies on the inpatient psychiatric wards at Eastbourne Department of Psychiatry. We ran a series of 5 scenarios, covering: cardiac arrest, ligature, lacerations, anaphylaxis, and sepsis, and involved a diverse team of nurses, support workers and doctors. They were required to make an initial assessment, provide emergency treatment, and escalate as appropriate. Afterwards we led a debrief of the scenario, and identified key learning points for the group.

Most simulation training is delivered in non-clinical settings or a standardised clinical environment, which is effective for learning processes, but can be challenging when clinicians manage emergencies in the unique environment of the psychiatric hospital. Our training took place in different locations within the hospital, and a key element was requiring staff to make optimal use of the ward environment, and identifying the most effective means of gathering emergency equipment and medical support, all of which vary between sites.

**Objectives:**

We aimed to improve response to physical health emergencies in the psychiatric hospital. This was a high-fidelity simulation using the real ward environment, full body mannequin, props for injuries, and equipment identical to that found on the wards: crash-bag, oxygen cylinders, anaphylaxis kit.

**Methods:**

Scenarios were chosen based on those emergencies which the staff had least confidence in managing in the ward setting, and they were written in consultation with the local resuscitation leaders.

We gathered data on knowledge of the location of emergency equipment, how to summon an emergency medical response, and self-reported confidence in managing different scenarios before and after the training, on a 5 point Likert scale (1=high confidence, 5=low confidence), and free text feedback.

**Results:**

With n=11 and Wilcoxon Signed-Rank test, average reported confidence in managing lacerations improved from 3.7 to 2.7 (p=0.006); in anaphylaxis improved from 4.0 to 2.3 (p=0.005); in sepsis from 3.2 to 2.1 (p=0.018); and in cardiac arrest from 3.1 to 2.0 (p=0.02).

There was already full awareness of where to locate the crash bag and how to summon the emergency medical team.

Written feedback expressed a desire for regular sim training to consolidate the learning and improve confidence, and a wider set of emergency scenarios. A key theme in debrief was difficulty providing treatment in a cramped environment, positioning of the team and patient, and identifying a clear team leader.

**Conclusions:**

We found an improvement in confidence across all scenarios, and the debrief was widely identified as helpful to consolidate learning. Further work would involve repeating the training to see if the improvement was maintained, and expanding to different sites across the trust.

**Disclosure of Interest:**

None Declared

